# Physical Activity Interventions for Patients With Poststroke Fatigue: Protocol for a Scoping Review

**DOI:** 10.2196/80703

**Published:** 2025-12-03

**Authors:** Dongling Chen, Lili Liang, Lei Ji, Xiao-Juan Guo

**Affiliations:** 1 Nanyang Institute of Technology Nanyang China; 2 Nanyang Central Hospital Nanyang China

**Keywords:** stroke, fatigue, scoping review, poststroke fatigue, physical activity, exercise, rehabilitation, protocol, knowledge synthesis, scoping literature review

## Abstract

**Background:**

Poststroke fatigue (PSF) affects nearly 50% of stroke survivors, severely impacting functional recovery and quality of life. Physical activity (PA) interventions show promise in mitigating fatigue, yet evidence remains fragmented across study designs and intervention types.

**Objective:**

This scoping review aims to systematically map the literature on PA interventions for PSF, identifying key concepts, evidence gaps, and implementation characteristics to guide future research.

**Methods:**

Using a 5-step framework, we will search PubMed, Web of Science, Scopus, Embase, PsycINFO, CINAHL, and the Cochrane Library from the inception of the databases to 2025. Gray literature and trial registries will be included. Two reviewers will independently screen titles, abstracts, and full texts using predefined criteria. Data extraction will focus on intervention components, fatigue assessment tools, and implementation outcomes.

**Results:**

Initial searches identified 8268 articles. The study selection process is expected to finish in February 2026, and the manuscript will be submitted in June 2026.

**Conclusions:**

The conduct of this scoping review is expected to synthesize a fragmented evidence base, providing a clear evidence map of PA interventions for PSF. The findings will identify key gaps and inform the design of future definitive studies and evidence-based clinical guidelines, ultimately contributing to improved care for stroke survivors experiencing fatigue.

**International Registered Report Identifier (IRRID):**

DERR1-10.2196/80703

## Introduction

Stroke refers to a group of cerebrovascular diseases characterized by sudden onset and rapidly developing focal or diffuse neurological deficits caused by organic brain injury [1]. Stroke is the second leading cause of death worldwide, accounting for 11.8% of global mortality [2-4]. It is marked by high incidence and mortality rates, significantly impairing patients’ quality of life [5]. Its complications significantly impact patients’ long-term functional recovery [6], with poststroke fatigue (PSF) being one of the most prevalent and direct contributing factors.

PSF is defined as a multidimensional sensorimotor, affective, and cognitive experience characterized by early-onset exhaustion and a lack of energy, which manifests as a feeling of weariness during physical or mental activity and cannot be alleviated by rest [7]. A meta-analysis [8] has identified 5 core characteristics of PSF: lack of physical strength to perform activities, abnormal need for prolonged sleep, easy fatigability after activity and abnormal need for rest, unexplained and unpredictable fatigue sensation, and increased stress sensitivity. These symptoms must be prominently present daily or frequently during at least 2 weeks within the past month.

PSF is a pathological, chronic, and persistent syndrome of physical and mental exhaustion experienced by stroke survivors [9]. Different studies have reported markedly divergent prevalence rates for PSF, ranging from 23% to 85% [10]. This significant heterogeneity primarily stems from some key factors. Foremost, the absence of standardized clinical diagnostic criteria and validated assessment scales for PSF results in substantial prevalence variations across studies using different measurement tools [11]. As a recent systematic review and meta-analysis [12] reported, the global pooled prevalence of PSF in stroke survivors was 46.79%.

PSF directly impedes stroke rehabilitation progress and severely compromises patients’ quality of life [13,14]. A prospective cohort study [15] found that PSF is a factor associated with the inability to return to work in patients younger than 60 years. PSF also significantly influences every domain of health-related quality of life and increases the caregiving burden placed on families and society [16]. Despite its high prevalence and substantial impact, the underlying mechanisms of PSF remain poorly understood, and effective management strategies are limited. However, insufficient attention has been paid to patients with PSF in clinical practice, which directly affects patient prognosis.

Current PSF management strategies consist of pharmacological and nonpharmacological interventions, with nondrug approaches being the principal method due to limited evidence supporting medication efficacy [17]. Bivard et al [18] suggested that PSF can be alleviated through pharmacological treatment, whereas Poulsen et al [19] found that medication has no effect on PSF. De Doncker et al [20] administered transcranial direct current stimulation to patients with PSF, resulting in marked improvement in their fatigue. Nguyen et al [21] concluded that cognitive behavioral therapy can improve fatigue symptoms more effectively than routine treatment among patients who have experienced a stroke. To date, evidence regarding the effectiveness of any intervention to treat or prevent PSF is still lacking [22]. Meanwhile, multiple studies [22,23] have suggested physical activity (PA) as a nonpharmacological intervention for PSF; however, this topic has not been systematically investigated in a review.

PA is a simple and easily implementable intervention for managing PSF. The American Heart Association and American Stroke Association’s statement [24] for health care professionals notes that PSF may be exacerbated by a sedentary lifestyle, and regular PA may help reduce PSF. Previous studies [25-27] have indicated that PA level is a determinant of PSF, with higher levels of PA associated with reduced fatigue severity. A meta-analysis [28] also revealed associations between higher PSF and lower PA. A pilot study [29] found that mobile health–based interventions have the potential to improve fatigue in patients who have experienced stroke. Exercise may improve several peripheral factors, such as decreased aerobic capacity and muscle atrophy, and central system dysregulations, including changes in cerebral blood flow, altered cellular energy reserves, and abnormal neural circuit activity, all of which could potentially contribute to PSF [30,31].

PA interventions have shown promise in managing fatigue in various clinical populations, including cancer survivors [32-34] and individuals with chronic fatigue syndrome [35,36]. However, the evidence for the effectiveness of PA interventions specifically targeting PSF is fragmented and has not been systematically synthesized. The heterogeneity in exercise interventions and outcome assessments precludes a systematic review, making a scoping review the most appropriate alternative. A scoping review is an ideal methodology to map the existing literature, identify gaps in knowledge, and provide a comprehensive overview of the types of evidence available. This scoping review protocol outlines a systematic approach to examining the scope and nature of PA interventions for PSF. Therefore, the purpose of this scoping review is to map PA intervention characteristics, delivery methods, and outcomes reported in PSF research.

## Methods

### Study Design

The scoping review will be conducted following the methodological framework proposed by Arksey and O’Malley [37], which includes the following five stages: (1) identifying the research question; (2) identifying relevant studies; (3) selecting studies; (4) charting the data; and (5) collating, summarizing, and reporting the results. This protocol has been designed in accordance with the PRISMA-ScR (Preferred Reporting Items for Systematic Reviews and Meta-Analyses Extension for Scoping Reviews) [38]. The PRISMA-ScR guidelines have informed the structure and reporting of this protocol and will guide the conduct and final reporting of the full review. As part of the scoping review process, we follow the guidelines set by the Joanna Briggs Institute [39]. Open Science Framework has registered the protocol for the study, which will begin in December 2025 and end in February 2026.

### Review Questions

Following preliminary literature screening, the research team established the study question through collaborative team discussions and specialist consultation. The primary research questions guiding this scoping review are as follows: (1) What are the characteristics of the PA interventions (eg, type, frequency, intensity, duration, session length, and total weeks) that have been used to manage PSF? (2) What is the volume and nature of the available evidence (eg, study designs and methodological quality), and what subpopulations of stroke survivors have been included in these studies? (3) What are the reported effects of PA interventions on fatigue severity and related domains (eg, physical function, mood, and quality of life), and what measurement tools are used to assess these outcomes? (4) On the basis of the mapping of existing evidence, what are the implications for developing optimal PA interventions and for designing future definitive trials for PSF?

### Ethical Considerations

Ethics approval is not required because this review will use secondary data synthesis without primary data collection. Furthermore, all analyzed data originate from publicly accessible sources, thereby eliminating confidentiality concerns and ensuring compliance with data protection regulations. Future dissemination of this work will include presentations at scientific meetings and publication in peer-reviewed journals.

### Search Strategy

A comprehensive search strategy will be developed in consultation with a medical information specialist. We will search the following electronic databases: PubMed, Web of Science, Scopus, Embase, PsycINFO, CINAHL, and the Cochrane Library. The search strategy will include a combination of Medical Subject Headings (MeSH) terms and keywords related to stroke, fatigue, and PA. Relevant subject headings and terms associated with the following concepts may be included in the search: (1) stroke, post stroke, poststroke, cerebral infarction, cerebral hemorrhage, hemorrhagic stroke, ischemic stroke, brain ischemia, brain infarction, and cerebrovascular accident; (2) fatigue, asthenia, lassitude, lethargy, tired, weak, and exhaust; and (3) sport, exercise, PA, movement, physical exercise, aerobic training, resistance training, exercise rehabilitation, walk, and step counts.

A Boolean search strategy (using operators such as AND and OR) will be used to merge these terms into queries optimized for specific databases or search engines. Reference lists of relevant articles will also be hand searched to identify additional studies. To ensure the validity and scientific rigor of the search strategy, we first conducted a preliminary search in PubMed, restricting the search terms to titles and abstracts while incorporating both keywords and MeSH terms. During the formal search phase, the search queries constructed in PubMed were refined and subsequently adapted for use in other databases to enable a comprehensive literature retrieval. The complete search strategy can be found in Multimedia Appendix 1. The search period will cover the period from the inception of the databases to December 31, 2025.

### Study Selection

Following initial retrieval, records will be aggregated in EndNote (Clarivate) for reference management and deduplication [40]. The cleaned dataset will then be transferred to Covidence (Veritas Health Innovation Ltd), which will serve as the primary tool for managing study selection and documenting the review workflow [41]. Two reviewers will independently screen the titles and abstracts of the identified studies for potential inclusion. A satisfactory interrater agreement (κ≥0.80) is required in this process. Full-text articles will be retrieved for studies that appear to meet the inclusion criteria or where there is uncertainty. The same 2 reviewers will then independently assess the full-text articles for final inclusion. Any disagreements will be resolved through discussion or by consulting a third reviewer. The search results and study screening process will be fully summarized according to PRISMA-ScR [38].

### Eligibility Criteria

The population, concept, and context framework [42] will be used in this study to define the inclusion criteria, which is a structured approach used in evidence synthesis.

### Types of Participants

The study participants were rigorously selected based on the following inclusion criteria: (1) all individuals had received a formal clinical diagnosis of stroke, as confirmed through standardized diagnostic protocols; (2) participants were required to be aged ≥18 years at the time of enrollment, ensuring legal adulthood and capacity to provide informed consent; and (3) patients were diagnosed with ischemic stroke, hemorrhagic stroke, transient ischemic attacks, or minor stroke.

### Concept

Any type of structured PA intervention, including but not limited to aerobic exercise, resistance training, balance training, and functional mobility training, meets the criteria. Studies where PA is part of a multicomponent intervention will also be included if the PA component can be clearly identified. Given the diverse definitions and measurement tools for PSF, it is impossible to establish a single definitive standard from a global perspective [43]. Therefore, any literature will be included if it has clear diagnostic criteria and assessment for fatigue.

### Context

This study will have no restrictions on language or publication date. All non-English literature will be translated into English, and then the results can be integrated. Interventions conducted in all health care and community settings will be included.

### Types and Sources of Literature

In addition to electronic databases, we will search gray literature sources, including Google Scholar, ClinicalTrials.gov, and ProQuest Dissertations.

### Exclusion Criteria

The exclusion criteria encompassed unavailable full-text articles despite exhaustive retrieval efforts, duplicate publications identified through author names, and datasets. Secondary literature, such as systematic reviews, narrative reviews, case reports, study protocols, opinion pieces, conference abstracts, commentaries, discussion papers, and publications with incomplete datasets, will also be excluded.

### Data Extraction

A data charting form will be developed to extract relevant information from the included studies. The form will be piloted on a small sample of studies and refined as necessary. Data to be extracted will include the following information:

- Study characteristics (author, year, country, and design)
- Participant characteristics (fatigue severity, sample size, age, gender, time since stroke, and severity of stroke)
- Intervention components (type, frequency, duration, intensity, setting, and technology use)
- Outcome measures used to assess fatigue and intervention results
- Implementation (dropout rate and safety events)
- Key findings related to the effectiveness of the intervention on fatigue

The complete data charting form can be found in Multimedia Appendix 2. Any discrepancies or challenges encountered during the data extraction process will be discussed within the research team. Revisions to the form will be made by consensus only when necessary to capture the data relevant to the review questions.

### Critical Appraisal of Individual Sources of Evidence

Notwithstanding that quality appraisal is not an essential step in scoping reviews, the included studies in this research will still be evaluated to ensure the robustness of the findings. Two reviewers will independently assess methodological quality of included studies using the enhanced Mixed Methods Appraisal Tool (MMAT). The decision to use the MMAT was motivated by the need to evaluate an anticipated combination of quantitative and qualitative evidence [44]. Its use of 5 core criteria ensures a methodologically rigorous yet efficient assessment. Any discrepancies in quality ratings will be resolved via consensus discussion. Consistent with the purpose of a scoping review to map the available evidence, no studies will be excluded based on quality assessment. Instead, the results of the MMAT appraisal will be used to characterize the overall methodological strengths and limitations of the evidence base. These findings will explicitly inform the interpretation of the review’s results, helping to contextualize the reported outcomes. Furthermore, identified gaps in methodological rigor will form the basis for recommendations for future primary research.

### Analysis and Reporting

The results of this scoping review will be reported in accordance with the PRISMA-ScR guidelines. Following data extraction using a standardized form, a narrative synthesis will be conducted and structured around a predefined analytical framework that directly addresses the research questions. This framework will organize the findings by key themes, namely the characteristics of the PA interventions (eg, type, dosage, and delivery), the properties of the available evidence base (eg, study designs and populations), and the reported outcomes alongside the measures used. Within this structure, quantitative data will be summarized descriptively, whereas any qualitative findings will be analyzed using systematic, inductive content analysis. The synthesized evidence will be presented through narrative summaries, tables, and figures, culminating in a descriptive summary that discusses the implications for clinical practice and future research while explicitly highlighting the identified gaps in the literature.

## Results

**Figure 1:**
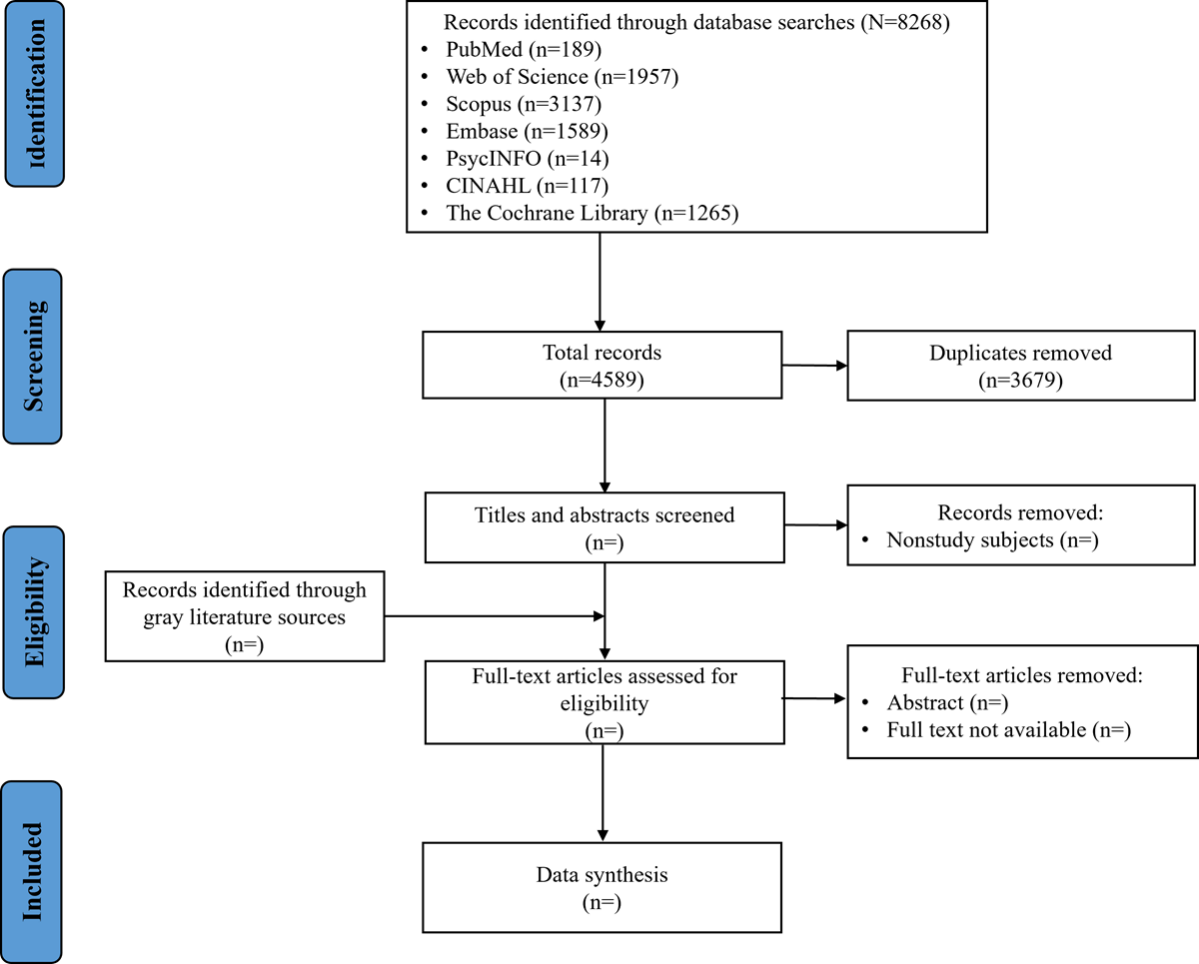
Flow diagram of study selection process.

A total of 8268 records have been identified through electronic database searches, and 4589 (55.5%) unique records remain after deduplication (Figure 1). As of November 2025, the literature screening process is still ongoing. Gray literature sources have not yet been searched. The study selection process is scheduled for completion in February 2026. Data analysis and manuscript writing are anticipated to be finalized by May 2026. Manuscript submission and peer review are expected to occur in June 2026.

## Discussion

### Anticipated Findings

Upon completion, this scoping review is anticipated to generate a systematic map of the existing literature on PA interventions for PSF. We hypothesize that our findings will reveal a heterogeneous and evolving evidence base, characterized by a wide variety of PA intervention types, dosages, and delivery modes. It is expected that the available studies will encompass diverse patient populations across different stroke recovery stages, using a range of fatigue assessment tools. A key anticipated finding is the identification of significant gaps in the literature, particularly regarding the optimal components and dosing of PA interventions for PSF, the mechanisms through which PA may alleviate fatigue, and the specific subpopulations of stroke survivors who may benefit most.

### Comparison With Prior Work

To our knowledge, this will be the first scoping review to systematically map the entire landscape of PA interventions specifically for PSF. While previous systematic reviews have focused on the general association between PA and fatigue or have examined interventions for PSF broadly, this review will provide a dedicated and detailed characterization of PA as a distinct intervention category. The findings will complement existing knowledge by offering a comprehensive overview of intervention characteristics and implementation contexts, which can inform the design of more targeted and effective future trials. This work will situate itself within the broader context of nonpharmacological PSF management, potentially clarifying the unique role of PA compared to other approaches such as cognitive behavioral therapy or neuromodulation.

The findings of this review are expected to directly inform future research by pinpointing specific evidence gaps. These may include the need for high-quality randomized controlled trials to determine optimal PA prescription, studies exploring the biological and psychological mechanisms of action, and research focused on underrepresented stroke subgroups. On the basis of mapped evidence, we will formulate concrete recommendations for the key components and design of such definitive trials.

### Strengths and Limitations

A strength of this scoping review is the comprehensive search strategy that includes multiple databases and gray literature sources, which will help to ensure that a wide range of studies are identified. The use of independent screening by 2 reviewers will enhance the reliability of the study selection process. In addition, the conduct of a meta-analysis was not feasible with the available quantitative data, which hindered the advancement of recommendations for an optimal exercise regimen. Finally, there may be potential challenges in synthesizing highly heterogeneous interventions.

### Conclusions

In conclusion, this scoping review protocol outlines a systematic approach to examining the scope and nature of PA interventions for PSF. However, the study implementation requires strict adherence to guidelines, ensuring rigor, preventing outcome bias, and addressing potential feasibility constraints. The execution of this review is expected to consolidate a currently fragmented body of literature, providing a valuable resource for researchers, clinicians, and policymakers. The findings will contribute to a better understanding of the current evidence base and inform future research and clinical practice in this important area of stroke rehabilitation.

## Appendix

Multimedia Appendix 1 Search strategy.

Multimedia Appendix 2 Data charting form.

## Abbreviations

MeSH: Medical Subject Headings
MMAT: Mixed Methods Appraisal Tool
PA: physical activity
PRISMA-ScR: Preferred Reporting Items for Systematic Reviews and Meta-Analyses Extension for Scoping Reviews
PSF: poststroke fatigue

## Data Availability

The datasets generated or analyzed during this study will be available from the corresponding author on reasonable request.
