# Supplementary material for: Physical Activity Interventions for Patients With Poststroke Fatigue: Protocol for a Scoping Review
Source: JMIR Res Protoc. 2025 Dec 3;14:e80703. doi: 10.2196/80703 (PMC12712566; doi:10.2196/80703)
Supplement: Multimedia Appendix 1 [file resprot_v14i1e80703_app1.pdf]

Supplementary material 1: Search strategy

PubMed

Search conducted: December 9, 2025

**Search**	**Query**	**Records retrieved**
#1	stroke[MeSH Terms]	195,447
#2	((((((((post stroke[Title/Abstract]) OR (post-stroke[Title/Abstract])) OR (cerebral infarction[Title/Abstract])) OR (cerebral hemorrhage[Title/Abstract])) OR (hemorrhagic stroke[Title/Abstract])) OR (ischemic stroke[Title/Abstract])) OR (brain ischemia[Title/Abstract])) OR (brain infarction[Title/Abstract])) OR (cerebrovascular accident[Title/Abstract])	137,225
#3	#1 OR #2	258,303
#4	fatigue[MeSH Terms]	42,328
#5	(((((asthenia[Title/Abstract]) OR (lassitude[Title/Abstract])) OR (lethargy[Title/Abstract])) OR (tired[Title/Abstract])) OR (weak[Title/Abstract])) OR (exhaust[Title/Abstract])	277,376
#6	#4 OR #5	318,583
#7	(((((((((sport[Title/Abstract]) OR (exercise[Title/Abstract])) OR (physical activity[Title/Abstract])) OR (movement[Title/Abstract])) OR (physical exercise[Title/Abstract])) OR (aerobic training[Title/Abstract])) OR (resistance training[Title/Abstract])) OR (exercise rehabilitation[Title/Abstract])) OR (walk[Title/Abstract])) OR (step counts[Title/Abstract])	897,926
#8	#3 AND #6 AND #7	189
